# A Dietary Intervention with Reduction of Starch and Sucrose Leads to Reduced Gastrointestinal and Extra-Intestinal Symptoms in IBS Patients

**DOI:** 10.3390/nu11071662

**Published:** 2019-07-20

**Authors:** Clara Nilholm, Bodil Roth, Bodil Ohlsson

**Affiliations:** Department of Internal Medicine, Lund University, Skåne University Hospital, 205 02 Malmö, Sweden

**Keywords:** diet, extra-intestinal symptoms, gastrointestinal symptoms, irritable bowel syndrome, starch, sucrose

## Abstract

Patients with irritable bowel syndrome (IBS) exhibit low-grade inflammation and increased gut permeability. Dietary sugar has been shown to contribute to low-grade inflammation and increased gut permeability, and to correlate with gastrointestinal (GI) symptoms. The aim of the present study was to examine the effect of a starch- and sucrose-reduced diet (SSRD) on gastrointestinal (GI) and extra-intestinal symptoms in IBS. One hundred and five IBS patients (82 women, 46.06 ± 13.11 years), with irritable bowel syndrome-symptom severity scale (IBS-SSS) > 175, were randomized to SSRD for 4 weeks or continued ordinary eating habits. The visual analog scale for irritable bowel syndrome (VAS-IBS), IBS-SSS, and 4-day food diaries were collected at baseline and after 2 and 4 weeks. After the intervention, one-third of the patients did not fulfill the criteria for IBS/functional gastrointestinal disorder. Half of the participants changed from moderate/severe disease to no/mild disease according to IBS-SSS. Comparisons between the groups showed decreased weight and sweet cravings, and parallel decreases in total IBS-SSS and extra-intestinal IBS-SSS scores, in the intervention group compared to controls (*p* < 0.001 for all). When calculating separate extra-intestinal symptoms, belching (*p* = 0.001), muscle/joint pain (*p* = 0.029), urinary urgency (*p* = 0.017), and tiredness (*p* = 0.011) were decreased after introduction of SSRD compared to controls. In conclusion, SSRD improves both GI and extra-intestinal symptoms in IBS.

## 1. Introduction

Irritable bowel syndrome (IBS) is a functional bowel disease without known etiology [1]. In epidemiological studies, an association has been found between IBS and irregular meal intake, fast food, and sweets [2,3,4,5,6]. It has recently been found that many patients with IBS have poor dietary habits, with irregular meal intake and high intake of cereals, sweets, and soft drinks, and a low intake of vegetables, fruits, and fish, with correlations between the intake of soft drinks and gastrointestinal (GI) symptoms [7]. Dietary sugar contributes to low-grade inflammation and increased gut permeability [8], characteristics often described in IBS [9]. One might speculate that the pathophysiology behind IBS depends on unhealthy dietary patterns, rather than a primary bowel disease, in a subgroup of patients.

IBS is associated with psychological disorders, and emotional factors affect the experience of pain [10,11]. These factors contribute to the characteristic visceral hypersensitivity observed in IBS [12]. Through afferent and efferent pathways between the gut and central nervous system, hypersensitivity may also be present in other visceral organs, so called cross-reactivity [13,14]. This may constitute one explanation for extra-intestinal symptoms and comorbidity of other pain conditions in IBS patients [1].

One of the first treatments of choice in IBS is dietary recommendations in the form of National Institute for Health and Care Excellence (NICE) guidelines or low fermentable oligo-, di-, mono-saccharides And polyols (FODMAP) diet. The effect of current recommendations is a reduction of GI symptoms in about 50% of the subjects [15,16]. However, the effect of the diet on extra-intestinal or associated symptoms has, to our knowledge, not been studied. Improved quality of life and psychological well-being have previously been found in type 2 diabetes after a dietary intervention with carbohydrate restrictions [17]. The starch- and sucrose-reduced diet (SSRD) [18] was administered to patients with IBS in a clinical trial, which was motivated by findings of increased prevalence of rare sucrase-isomaltase (SI) pathogenic variants in IBS patients [19,20]. A first report described a marked effect of SSRD on GI symptoms and psychological well-being in IBS patients after 2 weeks of treatment [7]. If the theory of cross-reactivity is true, extra-intestinal symptoms should also improve after a dietary intervention, together with the decreased GI symptoms.

Our hypothesis was that introduction of SSRD should reduce extra-intestinal symptoms along with the improvement of GI symptoms. To address this, 105 IBS patients from the southernmost district of Sweden were enrolled in a prospective dietary intervention study. Participants randomized to the dietary intervention group reduced their intake of starch and sucrose for 4 weeks, whilst those randomized to the control group continued with their normal diet. Questionnaires concerning GI symptoms, extra-intestinal symptoms and a 4-day food diary were completed at study start and after 2 and 4 weeks. The aim of the present study was to study the effect of SSRD for 4 weeks on overall GI symptoms, in parallel with psychological well-being and extra-intestinal symptoms, in IBS patients.

## 2. Material and Methods

The study was approved by the Ethical Review Board of Lund University (2017/171, 2017/192) and performed in accordance with the declaration of Helsinki. All subjects gave their written, informed consent before inclusion in the study. The study was registered at ClinicalTrials.gov data base (NCT03306381).

### 2.1. Patients

Patients between 18 and 70 years of age, who had received the diagnosis IBS using the International Statistical Classification of Diseases and Related Health Problems (ICD-10), K580 or K589, were recruited from all primary health care centers (PCC) in the southernmost district of Sweden (2015–2017) and one tertiary health care center (2016–2017). In total, 2034 IBS patients from the primary health care, and 789 IBS patients from the tertiary health care center, were identified. After exclusion of duplicates, 1679 patients remained. Invitation letters were sent to 679 patients after exclusion of all patients not fulfilling the inclusion criteria (Figure S1), which included subjects living outside the cities of Lund and Malmö or in their closest neighborhood, or an inability to find a telephone number for the subjects. Patients who were contacted and willing to participate (*N* = 145, 112 patients (77.2%) from PCC, 34 men (23.4%)) were sent a package of study questionnaires to complete prior an appointment at the Internal Medicine Research Group, Skåne University Hospital, Malmö. After initial agreement, 40 patients (27.6%), 11 men (27.5%), did not participate in the study because they did not show up or were not willing to participate at a later time point (*N* = 18), had mild symptoms (*N* = 14), wrong diagnoses (*N* = 5) or were already on a diet in the exclusion criteria (i.e., gluten-free diet, vegan diet, low carbohydrate and high fat (LCHF) diet or low FODMAP diet (N = 3). Thus, 105 patients (23 men (21.9%)) were finally enrolled in the study (77 patients (73.3%) from the PCC) from the 679 invitation letters sent (15.5% inclusion rate) (Figure S2). Reasons not to being available or declining study participation are presented in Table S1.

### 2.2. Study Design

The study design was a randomized, open clinical trial with a dietary intervention lasting 4 weeks (Figure S1). Patients with IBS were contacted via mail and thereafter telephone. After an agreement to participate in the study, patients completed a study questionnaire addressing socioeconomic factors, lifestyle habits, and a health declaration. Patients also completed a food diary during day 6–10 of the run-in period, the Rome IV questionnaire, the irritable bowel syndrome-symptom severity scale (IBS-SSS), and the visual analog scale for irritable bowel syndrome (VAS-IBS). At the first appointment, the questionnaires were studied and subjects who suffered from abdominal pain at least one day/week in combination with altered bowel symptoms and rated ≥ 175 scores of the IBS-SSS, without fulfilling any exclusion criteria (Figure S1), were included in the study. Anthropometric measurements of weight (kg) and height (m) in light in-door clothing, and systolic and diastolic blood pressures (mmHg) (Omron^®^ automatic reading) in supine position were performed at study start, as well as after the study completion. After randomization to dietary intervention, written and oral information about the diet was given. Patients randomized to serve as controls were informed to keep their ordinary food habits during the observational time, and were informed that they should receive all information about the diet after completion of the study. The dietary regimen was kept for 4 weeks, when patients had to complete a food diary during day 10–14 and 24–28; IBS-SSS and VAS-IBS after 2 and 4 weeks; and Rome IV after 4 weeks. The degree of sweet cravings before study start and after 4 weeks was estimated on a visual analog scale. The study started in January 2018 and was completed in February 2019.

### 2.3. Dietary Advice

The patients were instructed by one of the two physicians, CN or BO, to hold a diet with starch and sucrose restriction, according to the advice given to patients with congenital sucrase-isomaltase deficiency (CSID) [18]. Information from the webpage was translated into Swedish and given in written, along with the oral description. Briefly, all forms of sucrose-containing food, e.g., sweets, cakes, jam, and juice, were to be avoided. The content of starch should be reduced with less intake of cereals, but more intake of meat, fish, egg, and dairy products. Lists of suitable fruits and vegetables with less starch content were given (Tables S2 and S3). The content of gluten and lactose in the ingested products were unrestricted. Fiber-rich bread, raw rice, and fiber-rich pasta were preferred instead of white bread and ordinary rice and pasta, to delay the nutrient transport through the GI tract. Adding fat and/or protein to starch-rich foods was also recommended, in order to further delay GI transport and enhance starch tolerance through longer exposure to digestive intestinal enzyme activity in the small intestine. The patients were encouraged to eat slowly and chew their food properly, to increase the secretion of amylase, which can contribute to degradation of starch. Sweets and cakes were to be replaced with nuts in the case of sweet cravings. The only advice encouraged were regarding starch and sucrose restrictions, and how to enhance the tolerability of these compounds when ingested. They were not instructed to reduce their energy content or change overall dietary habits such as meal frequencies or regularity. The participants were encouraged to continue with their ordinary degree of physical activity and ordinary medications, without making any changes. If they used any form of probiotics or were on a diet not excluding them from enrollment, they should continue with this during the study, without introducing any new drugs or other dietary changes. The participants could reach the study staff by telephone or email, whenever they wanted during the study. The controls were encouraged to keep their ordinary dietary habits throughout the 4-week period. At the second meeting at week 4, they received the same instructions about the diet as the intervention group received at baseline.

### 2.4. Questionnaires

#### 2.4.1. Study Questionnaire

A study questionnaire about sociodemographic factors, family history, lifestyle habits, medical health, and pharmacological treatment was completed prior to study start.

#### 2.4.2. Food Diary

All liquid and solid food intakes were registered during the 4 days (Wednesday to Saturday) prior to start of the study, in the middle of the intervention at day 10–14, and at the end of the intervention at day 24–28. Based on the diary registrations, meal patterns were divided into a regular dietary habit defined as 3–6 meals a day ingested at regular time points from day to day, or an irregular dietary habit when less than 3 meals or more than 6 meals were ingested daily, or when the meals were ingested at different time points from day to day.

#### 2.4.3. Rome IV Questionnaire

The Rome IV questionnaire has been developed to diagnose functional gastrointestinal disorders (FGID) [21]. Questions No 40–48 in the Swedish version of the questionnaire was used, after having received license from The Rome Foundation, Inc. (Raleigh, NC, USA).

#### 2.4.4. Irritable Bowel Syndrome-Symptom Severity Scale

The IBS-SSS consists of four questions answered on visual analogue scales (VAS), where scores close to 0 mm suggest “no symptoms”, and scores close to 100 mm suggest “severe symptoms”. The questions regard abdominal pain, abdominal distension, satisfaction with bowel habits, and the impact of bowel habits on daily life. In addition, a fifth question ask about the number of days with abdominal pain in the last 10 days. The maximum achievable score is 500. Scores between 75 and 174 suggest mild disease, scores 175–299 suggest moderate disease, and scores ≥ 300 suggest severe disease [22]. A responder is defined as ≥ 50-point reduction of the total IBS-SSS score. A secondary endpoint was applied in the current study, with a responder defined as a 50% reduction in the total IBS-SSS score. In addition, IBS-SSS extra-intestinal symptoms such as nausea, difficulties to eat a whole meal, reflux, belching, headache, back pain, leg pain, muscle/joint pain, urinary urgency, and tiredness were estimated on VAS scales. The maximum achievable score is 500, after dividing the sum score with a factor of two [22].

#### 2.4.5. Visual Analog Scale for Irritable Bowel Syndrome

The VAS-IBS is a validated questionnaire used to investigate GI symptoms and psychological well-being [23]. The items are measured on VAS scales from 0 mm to 100 mm, where scores close to 0 mm suggest “no symptoms”, and scores close to 100 mm suggest “severe symptoms”. The values are inverted from the original format, where lack of symptoms was set to 100. The questionnaire is also validated to measure changes of symptoms over time [24]. The item psychological well-being has been found to strongly correlate to positive and negative aspects of psychological well-being, anxiety in close relations, self-esteem, and coping skills [25].

#### 2.4.6. Sweet Cravings

The degree of sweet cravings was estimated on a VAS scale, where scores close to 0 mm suggest “no cravings”, and scores close to 100 mm suggest “severe cravings” [15].

#### 2.4.7. Statistical Analyses

Two hypotheses were raised: (1) Reduced dietary intake of starch and sucrose improves GI symptoms in the patient cohort and (2) reduced dietary intake of starch and sucrose improves extra-intestinal symptoms in the patients. The primary endpoint was a ≥ 50-point reduction of total IBS-SSS scores at week 4. No power analysis was performed since studies of SSRD in IBS have not been performed previously. However, in a previous nutrition study carried out by our research team where patients with type 2 diabetes received a carbohydrate-reduced diet, 23 patients were enough to demonstrate improved GI symptoms [17]. The distributions of values were tested by Kolmogorov–Smirnov test. Age, body mass index (BMI), and disease duration were normally distributed, and calculations to examine differences in these variables between groups were performed by Student’s t test. Some of the symptom scores and the dietary intake values were not normally distributed, and these parameters were therefore calculated by Spearman’s correlation test and Mann–Whitney U test. Comparisons of dichotomous variables were calculated by Fisher’s exact test. When comparing GI and extra-intestinal symptoms before and after dietary intervention, the Wilcoxon’s test was used. The differences of symptoms between 2 weeks and baseline, and 4 weeks and baseline, were calculated, and delta values were compared between groups. The differences of dietary intakes between 2 weeks and baseline were calculated, and delta values of intakes of cereals and sweets/soft drinks were compared with delta values of symptom differences in the same time interval. The intakes of cereals and sweets/soft drinks, and the total IBS-SSS and total extra-intestinal IBSS-SSS scores, were divided into higher or lower levels by the median value, to evaluate whether these factors affected the symptom response, measured as responders and non-responders. Values are presented as number and percentage, mean ± standard deviation, or median and interquartile range (IQR). Two-sided tests were used and *p* < 0.05 was considered statistically significant. All calculations were performed in SPSS (version 24; IBM Corporation, New York, US) and performed as intention-to-treat analysis.

## 3. Results

### 3.1. Basal Characteristics

In total, 105 patients (82 women, 78.1%) were included. The mean age was 46.06 ± 13.11 years, range 26–70 years. Mean BMI was 21.67 ± 3.94 kg/m^2^ (range 13.04–33.23 kg/m^2^). The disease duration was 19.88 ± 13.77 years. Concomitant diseases and current drug treatment are shown in Table S4. Eighty-eight patients (83.8%) from the study cohort had previously tried a diet. At the time point for inclusion in the study, 68 patients (64.8%) were on a current diet, such as lactose-free (*n* = 40, 38.1%), gluten-reduced (*n* = 25, 23.8%), or vegetarian (*n* = 12, 11.4%) diet. Probiotics was used by 17 participants (16.2%), four (16.0%) in the control group and 13 (16.3%) in the intervention group.

There were no differences in sex (22 women/3 men versus 60 women/20 men; *p* = 0.242), age (41.4 ± 14.5 versus 47.5 ± 12.4 years; *p* = 0.064), or BMI (20.4 ± 3.9 versus 22.1 ± 3.9 kg/m^2^; *p* = 0.064) distribution between the control and intervention group. The degree of physical activity was equal between groups (p = 0.838), and the most common physical activity was >120 min exercise each day (24.8%), followed by <30 min/day (22.9%) and 30–60 min/day (15.2%). The dietary patterns in the two groups were equal at baseline regarding type of food intake and meal regularity. At week 2, the intakes of fish, vegetables/legumes, fruits/nuts, and dairy products were higher in the intervention group compared to controls, with concomitant lower intake of cereals, including bread, pasta, and breakfast cereals, and sweets/soft drinks, including candies, cakes, ice cream, and sugar-rich soda (Table 1). The changes of intakes of fruits/nuts (*p* < 0.001) and dairy products (*p* < 0.001) were significantly increased, whereas the intakes of cereals (*p* = 0.001) and sweets/soft drinks (*p* < 0.001) were decreased, in the intervention group compared to the control group. The distribution of irregular food intake in the control group (n = 12, 48.0%) and intervention group (n = 40, 50.0%) did not differ at baseline (*p* = 1.00). After 4 weeks, fewer patients in the intervention group had irregular dietary habits than at baseline (n = 25, 31.3%; *p* < 0.001), as well as in the control group (n = 9, 36.0%; *p* = 0.002), a difference which was not statistically significant in comparison (*p* = 0.614).

### 3.2. Disease Classification

After classification according to Rome IV, 86 patients (81.9%) fulfilled the criteria of IBS, whereas 17 patients (16.2%) suffered from FGID, because of lack of a close association between the pain and altered bowel habits (i.e., two or more of the following at least in 30% of the time; pain associated with improvement or worsening with defecation, changed consistency of stool, or changed frequency of defecation), when strictly following the Rome IV criteria [1]. There were no differences in the distribution of IBS and FGID between the control group and the intervention group at baseline, whereas 23 participants in the intervention group did not fulfill any of the criteria for IBS/FGID after 4 weeks on SSRD (Table 2). Only patients with moderate or severe IBS/FGID were included in the study, but after the dietary intervention, 39 participants (48.8%) in the intervention group had no or mild disease compared to two participants (8.0%) in the control group (*p* < 0.001). The distribution of subgroups of IBS did not differ between treatment groups (Table 2).

After 4 weeks, seven subjects (28.0%) in the control group had a reduction of ≥50 points in the total IBS-SSS score compared to baseline, whereas the corresponding figure in the intervention group was 57 subjects (71.2%) (*p* < 0.001). When considering a responder as ≥50% reduction of the IBS-SSS score, 35 subjects (43.8%) of the intervention group fulfilled the criteria, whereas no one in the control group reached a 50% reduction (*p* < 0.001). Those with a gluten-reduced diet did not have a higher prevalence of responders according to a 50-point improvement of total IBS-SSS scores (*p* = 0.624), responders according to 50% reduction of total IBS-SSS scores (*p* = 0.456) or absence of FGID/IBS diagnosis after 4 weeks (*p* = 0.665). Similarly, those with a lactose-free or vegetarian diet did not have a different effect of SSRD compared with those without these diets (*p* = 1.000 for all, except *p* = 0.634 for lactose-free diet and absence of FGID/IBS diagnosis after 4 weeks).

### 3.3. Anthropometric Parameters and Sweet Cravings

**Figure 1:**
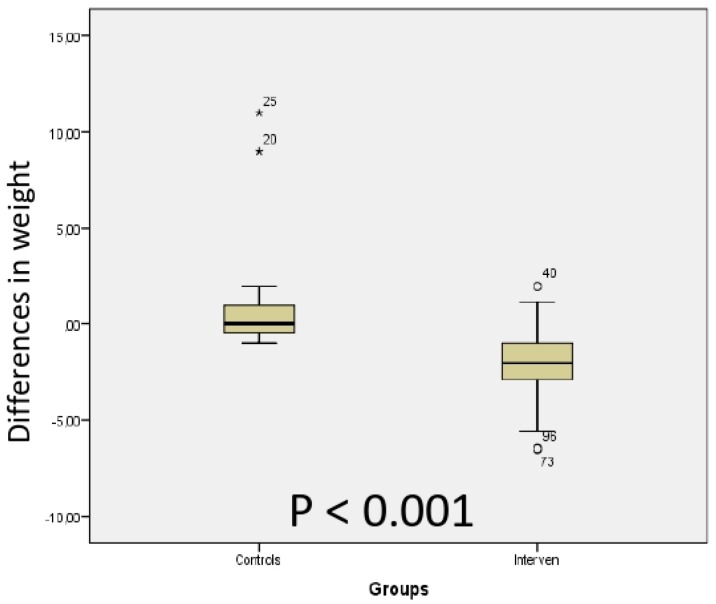
Comparison between the control and intervention group of the differences in weight between week 4 and baseline after introduction of a starch- and sucrose-reduced diet. Mann–Whitney U test. *P* < 0.05 was considered statistically significant.

The body weight was decreased after 4 weeks in the intervention group, but was unaffected in the controls (Table 3), with a significant difference in delta values between the groups (Figure 1). Neither systolic nor diastolic blood pressures were influenced by the dietary regime (Table 3). Sweet cravings was decreased in the intervention group (Table 3), which differed from the control group when comparing delta values (*p* < 0.001).

### 3.4. Gastrointestinal and Extra-Intestinal Symptoms

**Figure 2:**
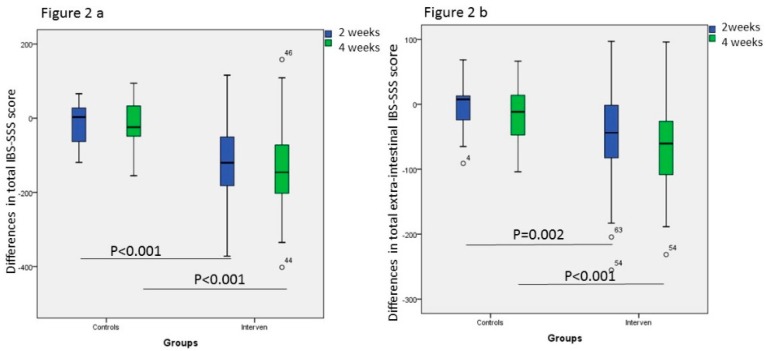
Comparison between the control and intervention group of the differences in (**a**) total irritable bowel syndrome–symptom severity scale (IBS-SSS) and (**b**) total extra-intestinal IBS-SSS scores between week 2 and baseline, and week 4 and baseline, after introduction of a starch- and sucrose-reduced diet. Mann-Whitney U test. *P* < 0.05 was considered statistically significant.

**Figure 3:**
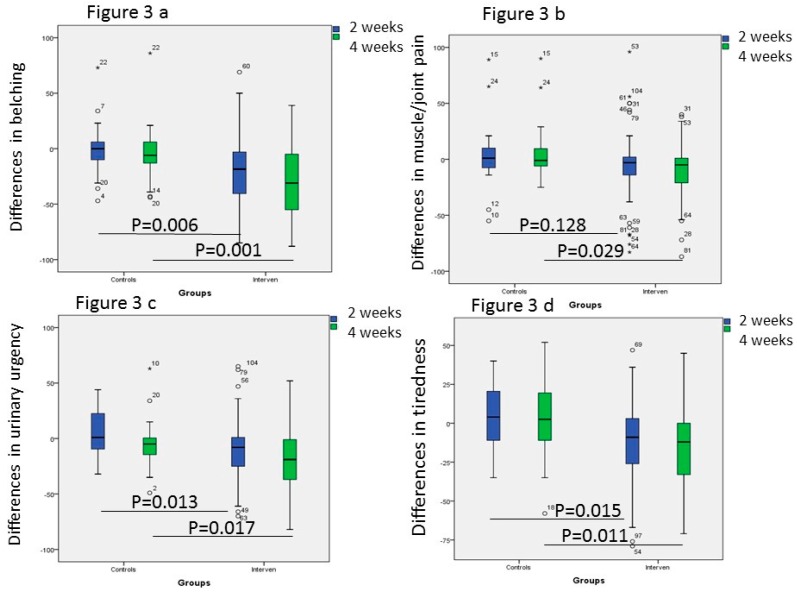
Comparison between the control and intervention group of the differences in (**a**) belching, (**b**) muscle/joint pain, (**c**) urinary urgency, and (**d**) tiredness between week 2 and baseline, and week 4 and baseline, after introduction of a starch- and sucrose-reduced diet. Mann-Whitney U test. *P* < 0.05 was considered statistically significant.

There were no differences in the total IBS-SSS scores, psychological well-being and the total extra-intestinal IBS-SSS scores, or individual extra-intestinal symptoms, between the control and intervention groups at study start (Table 4). After introduction of the SSRD, both intestinal symptoms measured as total IBS-SSS scores, psychological well-being, and extra-intestinal symptoms, except leg pain, were markedly improved already after 2 weeks. No one of the symptoms were improved in the control group (Table 4). When comparing differences in values between 2 weeks and baseline, and 4 weeks and baseline, i.e., delta values, there were statistically significant differences in total IBS-SSS score, total extra-intestinal IBS-SSS score, belching, urinary urgency, and tiredness between the control and intervention groups, whereas delta values of muscle/joint pain only was different after 4 weeks (Figure 2 and Figure 3). The improvements in total IBS-SSS scores after 4 weeks were correlated with total extra-intestinal IBS-SSS scores (*r* = 0.562, *p* < 0.001), psychological well-being (*r* = 0.358, *p* < 0.001), difficulties to eat a complete meal (*r* = 0.386, *p* < 0.001), belching (*r* = 514, *p* < 0.001), headache (*r* = 0.211, *p* = 0.040), muscle/joint pain (*r* = 215, *p* = 0.038), urinary urgency (*r* = 0.231, *p* = 0.025), and tiredness (*r* = 414, *p* < 0.001). When correlating the improvements in psychological well-being, this parameter was correlated with total extra-intestinal IBS-SSS scores (*r* = 0.407, *p* < 0.001), nausea (*r* = 0.288, *p* = 0.004), difficulties to eat a meal (*r* = 0.221, *p* = 0.031), reflux (*r* = 0.215, *p* = 0.034), headache (*r* =0.209, *p* =0.040), back pain (*r* = 0.212, *p* = 0.037), muscle/joint pain (*r* = 0.255, *p* = 0.01), and tiredness (*r* = 0.320, *p* = 0.001). 

Participants with a higher cereal intake were more prone to be responders (84.0%) than participants with lower intake (62.5%), according to a 50-point improvement of total IBS-SSS (*p* = 0.074), and according to a 50% improvement of total IBS-SSS (54.9% and 29.2%, respectively, *p* = 0.048). Higher or lower intake of sweets/soft drinks at baseline did not affect the amount of responders after 4 weeks (*p* = 0.473 and *p* = 0.781, respectively). Decreased cereal intake at week 2 was correlated with decreased total IBS-SSS scores (*r* = 0.315, *p* = 0.002) and belching (*r* = 0.344, *p* = 0.001) during the same time interval. The decreased intake of sweets/soft drinks was correlated with decreased total IBS-SSS scores (*r* = 0.244, *p* = 0.019), belching (*r* = 0.307, *p* = 0.003), and tiredness (*r* = 0.238, *p* = 0.022).

The degree or severity of symptoms could not predict which participants who should response to the SSRD (data not shown).

Those who had an effect of the SSRD, reported at the follow-up meeting that the effect was apparent already after a few days. One patient in the intervention group discontinued the study after 2 weeks because of more severe GI symptoms after introduction of the SSRD.

## 4. Discussion

The main finding of the present study was a marked reduction of weight, sweet cravings, and GI symptoms in IBS patients after introduction of SSRD, leading to that one-third of the cohort did not fulfill the Rome IV criteria for IBS/FGID after a 4-week dietary intervention. Further, half of the patients converted from moderate/severe disease to no or mild disease according to the IBS-SSS. Simultaneously, the total IBS-SSS score for extra-intestinal symptoms was reduced, with differences in belching, muscle/joint pain, urinary urgency, and tiredness between the control and intervention groups. The improvement in extra-intestinal symptoms was correlated with a decrease of the total IBS-SSS score and improved psychological well-being.

Overconsumption of dietary sugar has deleterious effects at both peripheral and central levels, including the regulation and secretion of hormones and neuropeptides [26], increased gut permeability [8], increased blood-brain barrier (BBB) permeability [8], low-grade inflammation [8], and dysregulation of the endocannabinoid system [27], the opioid system [28], and brain structures involving dopaminergic and rewarding systems [29]. Several of the effects of dietary sugar described above share similarities with pathogenetic mechanisms described in IBS [9]. This raises the hypothesis that our modern Western diet may play a causal role in the development of IBS/FGID. The loss of these diagnoses after SSRD, and the correlation between intake of soft drinks and GI symptoms recently described [7], support this hypothesis.

Sucrose intake leads to dopamine release, inducing a neuronal circuit promoting increased sucrose intake due to a rewarding value of sucrose [30]. Thus, not only a reduction of dietary calories due to a decrease of cereals and sweets/soft drinks into the lumen, but also disruption of the reward system leading to another eating behavior [30], may lead to more regular meal pattern with complete meals instead of frequent intake of sugar-rich products [7]. These mechanisms may explain the weight reduction and decreased sweet cravings after 4 weeks on the SSRD.

The GI symptoms evoked by excess intake of carbohydrates may depend on an overloading of the digestive system, with an accumulation of undigested carbohydrates in the lumen [31], which leads to gas production and osmosis with distention of the bowel [32,33]. The prompt effect of the SSRD on symptoms already after a few days, supports a luminal explanation for the effect [7]. Not only the dietary content was affected after introduction of the SSRD; many patients in the intervention group also had more regular meal patterns after introduction of the diet. The latter was, however, also found in the controls. A second explanation for reduced GI symptoms observed may be an alteration of the gut microbiota composition, since several studies have shown that the modern Western diet is associated with dysbiosis, which may lead to development of various diseases [34]. Third, genetic deficiency of sucrase-isomaltase activity may explain some of the improvements [19,20]. A genetic defect could be one cause of unclear GI symptoms, since the *SI* genes are responsible for a high degree of the enzymatic activity in the brush border of the enterocytes [35]. Fourth, the visceral hypersensitivity observed in IBS may be induced by peripheral, local or systemic, low-grade inflammation [12,36]. The positive effect of SSRD could be due to reduced inflammation after reduction of refined sugar content, since severe postprandial inflammation exerts adverse effects on health [37]. However, in a previous study examining this relationship, there were difficulties to associate the degree of inflammation with GI symptoms and psychological well-being [38].

The improvement of extra-intestinal symptoms may be explained by improved psychological well-being, since there is a close association between psychological factors and experience of pain [10]. In analogy, psychological therapies in the form of hypnotherapy, cognitive behavior therapy, and so on are amongst the most efficient treatment options for IBS [1,29].

Convergence of afferent pathways at the level of the spinal cord and at higher centers of the central nervous system contributes to the cross-reactivity of painful conditions from several different organs [14]. Both viscera-somatic and viscera-viscera convergence of sensory pathways are important and contribute to the coexistence of pain states affecting more than one organ [13,39]. A bidirectional cross-sensitization of the colon and urinary tract has been described in a rat model [13], possibly explaining the parallel improvement of GI and extra-intestinal symptoms observed. Since there is a great comorbidity between IBS and other pain conditions [1], it is of great importance not only to reduce GI symptoms, but also to reduce extra-intestinal symptoms.

Although gluten-sensitivity has been frequently described as an explanation to IBS symptoms, our participants still had IBS even though several had reduced their dietary gluten content. A recent systemic review found that the positive effects on IBS symptoms of gluten-reduction is not that obvious, when performed in randomized trials [40]. Rather, it may be the fructan content in wheat, and not the actual gluten content, that elicits symptoms [41]. It is possible that the reduced fructan content, rather than the reduced starch intake, was responsible for some of the effect observed in the present study with reduced intake of cereals. In the future, different mechanisms behind the effects evoked by the SSRD, including genetic analyses, have to be examined. If there is no concordance between genetic analyses of *SI* genes and improvement of symptoms, other nutrients such as fructan, have to be considered in the mechanistic analyses. It seems as though it is important to reduce the total burden of carbohydrates in the bowel lumen, preferably by restrictions of carbohydrates without any nutritional importance.

The present result of loss of the diagnosis in almost one-third of the intervention group raises the question about diagnostic accuracy. Previous research has described how physical activity is of importance in reducing GI symptoms and improving psychological well-being [42]. The poor dietary pattern described in this study cohort [7], as well as previous associations between IBS and physical activity [2,42], irregular meal intake, fast food, and candies [2,3,4,5,6], suggest that proper evaluation of lifestyle habits must be undertaken before the diagnosis of IBS/FGID is set. To improve lifestyle habits, and thereby reducing the diagnosis of FGID, would have great impact on society, due to the enormous disease burden and costs of an unhealthy lifestyle.

To succeed with a dietary intervention or lifestyle change, easy-to-follow dietary advice is important. For future studies, providing participants with menus and recipes in addition to giving dietary advice is highly recommended. As the issue of compliance always poses a challenge in dietary interventions, such strategies could be used in order to help participants to better adhere to the diet and vary their food composition. In the current study, the dietary advice was given by physicians. One can speculate whether the results had been different whether the advice had been provided by a dietitian. In a systemic review, dietary advice from a dietitian seemed to be more efficient than advice from a physician [43], but further studies are warranted for comparison.

The advantages of the SSRD is the rapid effect observed in those who experienced improvement of GI symptoms, and that the advice may be more simple to follow compared to other diets, with fewer food items to be eliminated. The greatest differences between SSRD and the low FODMAP diet is that the low FODMAP diet also recommends reduction of lactose intake and reduction of wheat and rye from cereals [32]. The incomplete effect of the low FODMAP diet may in some cases be explained by a focus on reduction of other carbohydrates than starch, e.g., some vegetables recommended in low FODMAP are rich in starch and not recommended in SSRD. There may be less risk of malnutrition after SSRD, since only starch and sucrose are intended to be excluded, and these items should be replaced by an increased amount of fish, vegetables, legumes, fruits, berries, and dairy products. However, all forms of diets may lead to elimination of important food items, and an unhealthy fixation of weight and food components.

There are several limitations of the present study. It is difficult to evaluate the participants’ compliance to the diet. However, the changed dietary habits in the intervention group suggest good compliance in most participants. The reduction of weight may indicate less energy intake, and does not necessarily mean that the participants have changed the nutrition composition. We have not assessed the change of FODMAP intake during the intervention, which is a limitation. It is also a limitation that we only have categorized the dietary intake at week 2, and not also on week 4. We think that the week 2 values better reflect the diet intake during the whole period, and have greater influence on symptoms and possible physiological processes than the intake during the last 4 days. In the next step, we will analyze the nutritional composition and portion amounts of the food consumed by each patient, excluding non-compliant participants from the intervention group. However, we then expect that the effect of the dietary intervention should be still strengthened, and that we rather underestimate than overestimate the SSRD benefits in the current report. The controls and active participants had the same amount of meetings with the investigators, and the same amount of questionnaires to complete, but the controls did not have any intervention. Therefore, some of the effects observed after the intervention may be due to a placebo effect, rather than a true dietary effect. We did not estimate differences in other lifestyle habits, work-related troubles or stress, which may also have influenced the study effect.

Regarding future directions, more studies concerning dietary sugar and dietary factors need to be evaluated in the pathogenesis of low-grade inflammation, increased gut permeability and development of IBS and FGID. Lifestyle habits must be evaluated in clinical practice, and improvement of symptoms after lifestyle changes should be properly evaluated before other treatments of functional conditions are considered. A clinical trial comparing the effect of low FODMAP and SSRD would be of great interest to perform.

In conclusion, almost one-third of participants did not longer fulfill the Rome IV criteria for IBS/FGID after introduction of SSRD for 4 weeks. Half of the participants changed from moderate/severe IBS/FGID to no/mild IBS/FGID according to the total IBS-SSS scores. In parallel with improved GI symptoms, total extra-intestinal symptoms were improved, especially belching, muscle/joint pain, urinary urgency, and tiredness.

## Figures and Tables

**Table 1:** Dietary intake before and after 2 weeks.

	Control Group *N* = 25	Differences within Group	Intervention Group *N* = 80	Differences within Group	*P*-Value
	Times/4 days		Times/4 days		
***Baseline*** Missing	1		2		
**Meat**	4 (1.25–8)		4 (2–8)		0.393
**Fish/seafood**	1 (0–1.75)		1 (0–2)		0.806
**Vegetable/legumes**	4 (2.25–4)		4 (3–4)		0.302
**Fruits/nuts**	2 (2–4)		3 (1–4)		0.652
**Dairy products**	4 (2–4)		4 (2–8)		0.604
**Cereals**	8 (4–8)		8 (4–8)		0.504
**Sweets/soft drinks**	4 (2–8)		4 (2–8)		0.920
***After 2 weeks*** Missing	3		6		
**Meat**	4 (2–7)	0 (−0.5–0.5)	4 (2–4)	0 (−2.25–0)	0.793
**Fish/seafood**	1 (0–1.25)	0 (−1–1)	1 (1–2)	0 (−2.25–1)	0.022
**Vegetable/legumes**	4 (2.5–5)	0 (0–1)	4 (4–8)	0 (0–4)	0.034
**Fruits/nuts**	2 (0.5–5)	0 (−1.5–1.5)	8 (4–8)	4 (0–6)	<0.001
**Dairy products**	4 (3–4)	0 (−1–0)	4 (3–8)	1 (0–4)	0.036
**Cereals**	8 (4–8)	0 (−0.5–0)	4 (1–8)	−4 (−6.25–0)	0.002
**Sweets/soft drinks**	4 (0.5–4)	0 (−1–0)	0 (0–2)	−3 (−4–1)	0.001

The control group continued with their ordinary food habits during the 2-week observational time period. The frequency of each food intake/day was registered, as well as the number of such days during the 4-day registration (day 6–10 during run-in and day 10–14 during the study). The total frequency of each item/4 days were compared between the two groups by Mann-Whitney U test. The intake differences between 2 weeks and baseline within each groups are shown. Values are presented as median and interquartile rages (IQR). *P* < 0.05 was considered statistically significant.

**Table 2:** Gastrointestinal symptoms in the cohort.

	Control Group *N* = 25	Intervention Group *N* = 80	*P*-Value
**Rome IV criteria**			
***Baseline***			
Unspecified FGID	6 (24.0)	11 (13.8)	0.352
IBS	19 (76.0)	67 (83.8)	
Mixed IBS	8	29	0.270 *
IBS-D	3	23	
IBS-C	7	13	
Unspecified IBS	1	2	
Missing value		2 (2.5)	
***4 weeks***			
No FGID/IBS		23 (28.8)	0.001
Unspecified FGID	7 (28.0)	9 (11.2)	
IBS	16 (64.0)	39 (48.8)	
Mixed IBS	5	10	0.815 *
IBS-D	5	17	
IBS-C	5	11	
Unspecified IBS	1	1	
Missing value	2 (8.0)	9 (11.2)	
**IBS-SSS** total score			
***Baseline***			
Moderate	11 (44.0)	37 (46.3)	0.821
Severe	14 (56.0)	41 (51.2)	
Missing value		2 (2.5)	
***4 weeks***			
<75		15 (18.8)	<0.001
Mild 75–174	2 (8.0)	24 (30.0)	
Moderate 175–299	9 (36.0)	24 (30.0)	
Severe ≥ 300	12 (48.0)	9 (11.9)	
Missing value	2 (8.0)	8 (10.0)	

FGID = Functional gastrointestinal disease, IBS = irritable bowel syndrome, IBS-D = diarrhea-predominated IBS, IBS-C = constipation-predominated IBS, IBS-SSS = irritable bowel syndrome – symptom severity scale. Values are presented as number and percentage. * = analyses of IBS subgroups. Fisher’s exact test. *P* < 0.05 was considered statistically significant.

**Table 3:** Anthropometric data and sweet cravings in the cohort.

	Control Group *N* = 25	*P*-Value	Intervention Group *N* = 80	*P*-Value
**Weight** (kg/m^2^)				
Baseline	68 (57–75) (0)		72 (64–85) (3)	
4 weeks	68 (61–76) (2)	0.158	71 (64–82) (9)	<0.001
**Systolic blood pressure** (mm/Hg)				
Baseline	127 (116–140) (1)		125 (115–135) (7)	
4 weeks	125 (120–148) (4)	0.974	120 (115–130) (8)	0.431
**Diastolic blood pressure** (mm/Hg)				
Baseline	80 (75–90) (1)		80 (70–85) (7)	
4 weeks	80 (75–85) (4)	0.222	80 (70–84) (8)	0.074
**Sweet cravings** (mm)				
Baseline	51 (34–70) (2)		60 (32–79) (10)	
4 weeks	57 (30–70) (3)	0.671	21 (10–42) (11)	<0.001

IBS-SSS = Irritable bowel syndrome–symptom severity scale. Sweet cravings were measured on a visual analog scale of 100 mm, where 0 was no cravings and 100 was maximal cravings. Values are presented as median (interquartile (IQR). Wilcoxon’s test. *P* < 0.05 was considered statistically significant.

**Table 4:** Symptom scores.

	Control Group *N* = 25	P-value	Intervention Group *N* = 80	P-value
**IBS-SSS Total Score**				
Baseline	310 (247–351)		306 (250–356) (2)	
2 weeks	271 (238–325) (5)	0.548	190 (118–282) (6)	<0.001
4 weeks	300 (233–331) (2)	0.248	156 (88–250) (8)	<0.001
**IBS-SSS Extra-Intestinal**				
Baseline	197 (106–257)		184 (125–254) (3)	
2 weeks	154 (121–241) (7)	0.823	117 (77–215) (6)	<0.001
4 weeks	169 (107–208) (3)	0.231	98 (61–174) (6)	<0.001
**Psychological Well-Being**				
Baseline	47 (24–71)		50 (24–69) (2)	
2 weeks	49 (31–66) (5)	0.658	41 (14–60) (6)	0.002
4 weeks	48 (32–60) (2)	0.732	36 (13–53) (6)	<0.001
**Nausea**				
Baseline	29 (6–50)		11 (1–34) (2)	
2 weeks	20 (3–38) (5)	0.820	4 (0–28) (6)	0.011
4 weeks	12 (2–56) (2)	0.112	2 (0–24) (6)	0.004
**Difficulty to Eat a Whole Portion**				
Baseline	9 (1–26)		12 (1–30) (2)	
2 weeks	8 (1–27) (5)	0.941	4 (0–22) (6)	0.003
4 weeks	6 (1–20) (2)	0.071	3 (0–17) (6)	<0.001
**Reflux**				
Baseline	17 (2–78)		16 (3–54) (2)	
2 weeks	14 (4–64) (5)	0.806	5 (1–23) (6)	<0.001
4 weeks	8 (1–50) (2)	0.061	4 (0–21) (6)	<0.001
**Belching**				
Baseline	67 (20–84)		71 (42–84) (3)	
2 weeks	64 (36–81) (5)	0.791	44 (15–60) (6)	<0.001
4 weeks	59 (27–78) (2)	0.246	26 (12–50) (6)	<0.001
**Headache**				
Baseline	31 (15–48)		23 (7–67) (2)	
2 weeks	30 (6–46) (5)	0.378	24 (5–48) (6)	0.009
4 weeks	25 (8–35) (2)	0.845	16 (4–36) (6)	0.001
**Back Pain**				
Baseline	45 (19–66)		37 (8–73) (2)	
2 weeks	34 (7–54) (5)	0.227	27 (6–53) (6)	0.007
4 weeks	32 (5–55) (2)	0.249	20 (1–42) (6)	<0.001
**Leg Pain**				
Baseline	3 (0–16)		4 (0–24) (2)	
2 weeks	4 (1–12) (6)	0.452	2 (0–16) (6)	0.178
4 weeks	4 (1–12) (2)	0.924	2 (0–16) (6)	0.006
**Muscle/Joint Pain**				
Baseline	21 (9–70)		44 (12–70) (2)	
2 weeks	25 (2–82) (3)	0.290	30 (6–64) (6)	0.022
4 weeks	47 (8–74) (2)	0.753	27 (6–50) (6)	<0.001
**Urinary Emergency**				
Baseline	20 (3–56)		49 (18–73) (2)	
2 weeks	21 (2–53) (6)	0.401	26 (4–64) (6)	<0.001
4 weeks	16 (1–43) (2)	0.131	21 (1–47) (6)	<0.001
**Tiredness**				
Baseline	67 (39–91)		65 (46–97) (2)	
2 weeks	70 (46–84) (5)	0.458	50 (26–73) (6)	<0.001
4 weeks	65 (41–83) (2)	0.794	48 (22–71) (6)	<0.001

IBS-SSS = Irritable bowel syndrome–symptom severity scale. Values are presented as median and interquartile rages (IQR). Wilcoxon’s test. *P* < 0.05 was considered statistically significant.

## Supplementary Materials

The following are available online at https://www.mdpi.com/2072-6643/11/7/1662/s1, Figure S1: Flow chart over inclusion and exclusion criteria, Figure S2: CONSORT 2010 Flow Diagram, Table S1: Reasons for not being eligible for/declining study participation, Table S2: Recommendations of fruit intake according to a starch- and sucrose-reduced diet, Table S3: Recommendations of vegetable and legume intake according to a starch- and sucrose-reduced diet, Table S4: Concomitant diseases besides irritable bowel syndrome (IBS) and drugs used as regular treatments.
