# Supplementary material for: A Dietary Intervention with Reduction of Starch and Sucrose Leads to Reduced Gastrointestinal and Extra-Intestinal Symptoms in IBS Patients
Source: Nutrients. 2019 Jul 20;11(7):1662. doi: 10.3390/nu11071662 (PMC6682926; doi:10.3390/nu11071662)
Supplement: Supplementary file 1 [file nutrients-11-01662-s001.pdf]

Supplementary Materials:

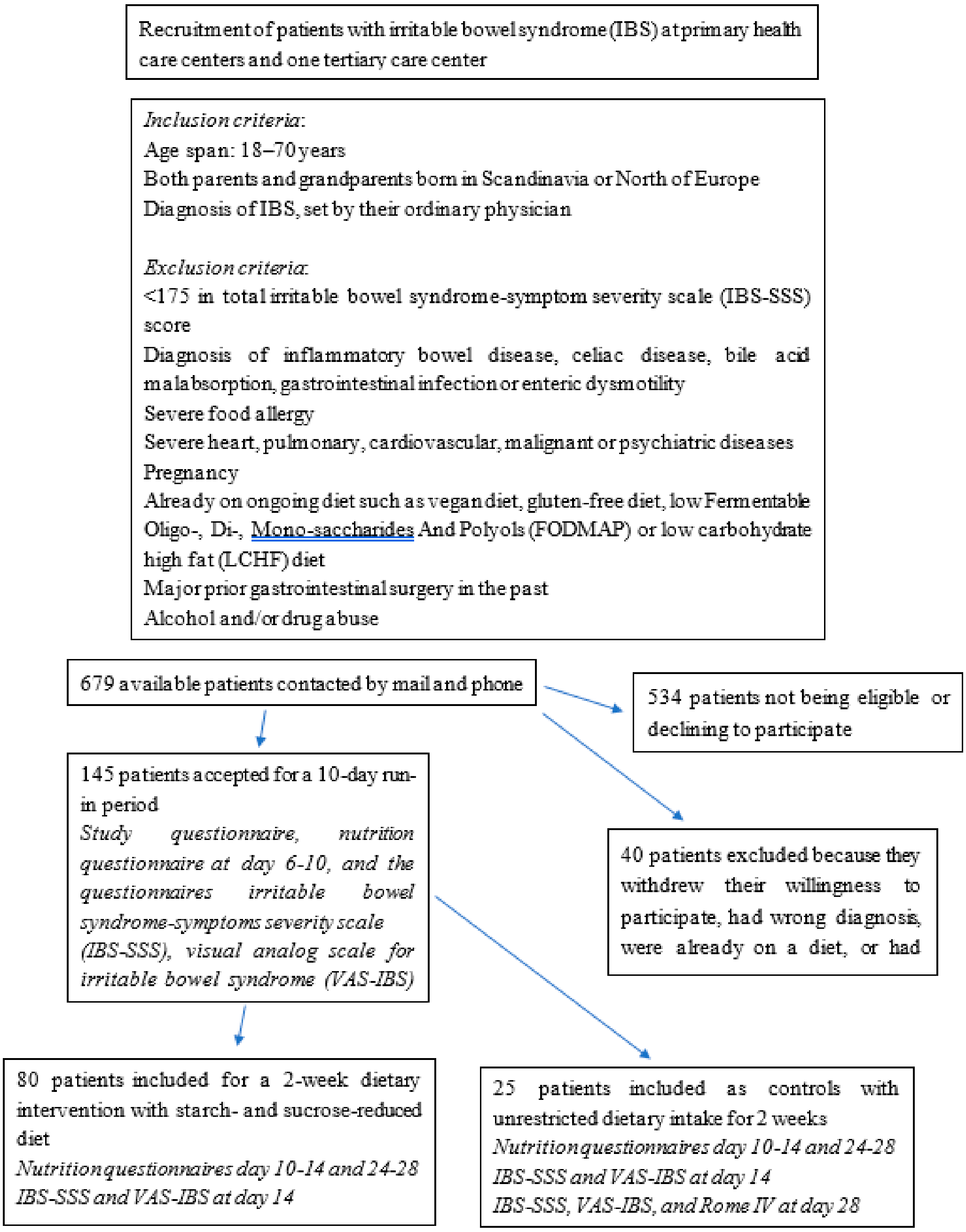

Figure S1: Flow chart over inclusion and exclusion criteria.

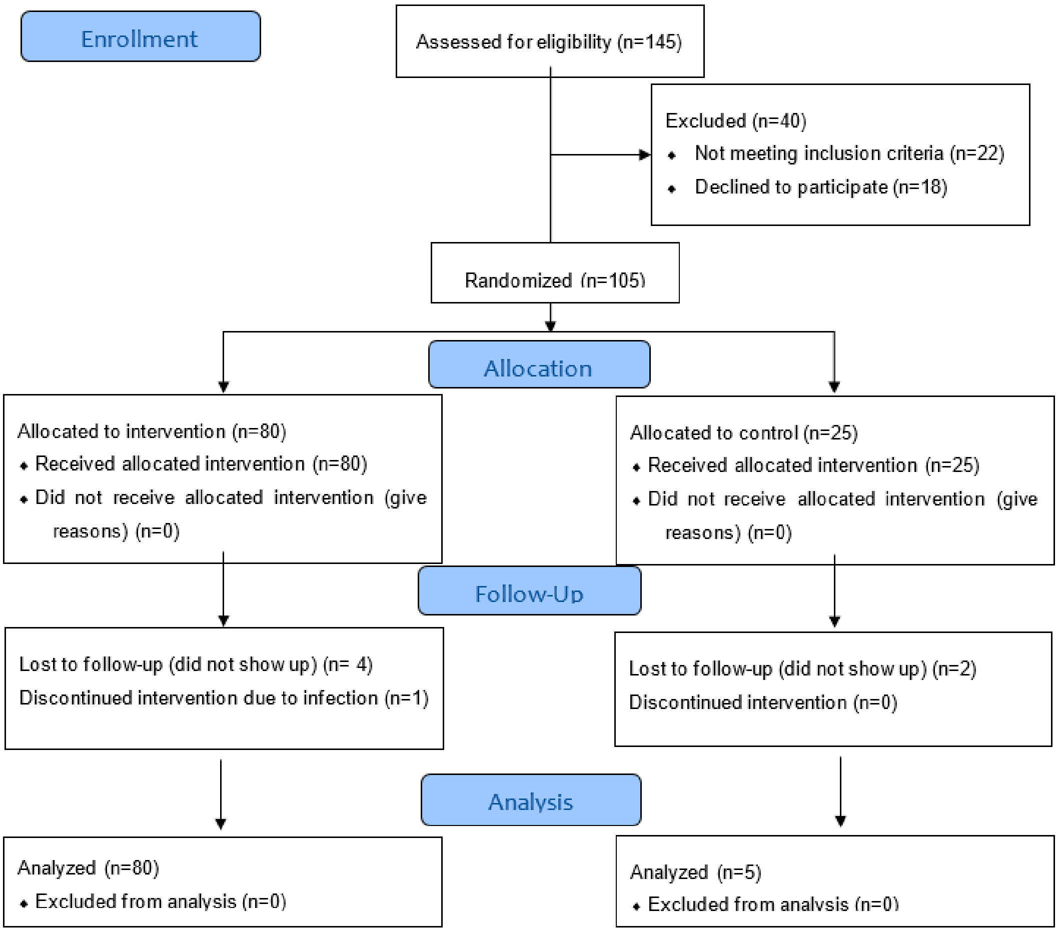

Figure S2. CONSORT 2010 Flow Diagram.

Table S1. Reasons for not being eligible for/declining study participation.

Reasons for Non-Inclusion	Number
Did not answer when calling	188
Did not want to participate	157
Wrong address	62
Already on a diet in the exclusion criteria	45
No or mild symptoms	37
Another diagnosis	27
Moved from the region	14
Pregnancy	4

Table S2. Recommendations of fruit intake according to a starch- and sucrose- reduced diet.

Well Tolerated	Tolerated by Some	Not Tolerated
Avocado	Persimmons	Apples
Blackberries	Plums	Apricots
Blueberries	Raisins	Bananas
Boysenberries	Watermelon	Cantaloupe
Cherries		Dates
Cranberries		Grapefruits
Currants		Guava
Figs		Honeydew melon
Gooseberries		Mangos
Grapes		Nectarines
Kiwi fruits		Oranges

Lemons	Passion fruits
Limes	Peaches
Loganberries	Pineapples
Olives	Tangelos
Papayas	Tangerines
Pears	
Pomegranates	
Prunes	
Raspberries	
Rhubarbs	
Strawberries	

Information from Ref No 9.

Table S3. Recommendations of vegetable and legume intake according to a starch- and sucrose-reduced diet.

Well Tolerated	Tolerated by Some	Not Tolerated
Alfafa sprouts	Edamame soybeans	Beets
Artichokes *	Jicamas	Black beans
Arugulas	Leeks	Black-eyed peas
Asparagus *	Okra	Butternut
Bamboo shoots	Pumpkins	Carrots
Bok choy	Snow peas	Cassavas
Broccoli *	Tempeh	Chickpeas
Brussels sprouts *	Tofu	Corn
Cabbages *	Yellow wax beans	Garlic
Cauliflower *		Green peas
Celery		Lentils
Chard		Kidney beans
Chicories		Lima beans
Chives		Navy beans
Collard greens		Onion
Cress		Parsnips
Cucumbers		Pinto beans
Eggplants		Potatoes
Endive		Soybeans
Green beans		Split peas
Kale		Sweet potatoes
Lettuces		Yams
Mung bean sprout		
Mushrooms		
Mustard green		
Peppers		
Radishes		
Spaghetti squash		
Spinach		
Tomatoes		
Turnips		
Yellow squash		
Zucchini		

Information from Ref No 9. * = These food items can evoke GI symptoms in all subjects.

Table S4. Concomitant diseases besides irritable bowel syndrome (IBS) and drugs used as regular treatments.

Diseases	
Allergy	17
Hypothyreosis	12

Asthma bronchialis	11
Depression	11
Hypertension	10
Migraine	7
Lactose intolerance	5
Pain in back and joints	5
**Drug Treatment**	
Antidepressants	21
Laxatives	14
Proton pump inhibitor	13
Levaxine	13
Hormonal treatment	11
Vitamin D	11
Statins	8
Folic acid	6
Cobalamin	5
Asthma inhalators	5
